# Tipped Over the Edge: A Case of Diabetes Ketoacidosis Precipitated by High-Dose Steroids in the Setting of a Large Peritonsillar Abscess

**DOI:** 10.7759/cureus.56917

**Published:** 2024-03-25

**Authors:** Marcelino Mederos Liriano, Katherine Hageboeck, Mark Colantonio, Saud Alawad, Jessica Thayer

**Affiliations:** 1 Internal Medicine, West Virginia University School of Medicine, Morgantown, USA

**Keywords:** dexamethasone, glucocorticoids, type 2 diabetes, type 1 diabetes, diabetic ketoacidosis

## Abstract

Peritonsillar abscess is an infection of tonsillar soft tissue which can spread into additional neck structures leading to symptoms of fever, sore throat, dysphagia, and airway compromise. We describe a case of diabetic ketoacidosis in a patient with a history of uncontrolled type II diabetes mellitus admitted for a peritonsillar abscess who received intravenous steroids for management of the abscess swelling. The patient was treated with an insulin drip, hydration, and electrolyte replacement with a resolution to his anion gap and metabolic acidosis. Diabetic ketoacidosis occurs during increased gluconeogenesis leading to ketosis and metabolic acidosis which can be a life-threatening condition if not quickly recognized and treated. This case highlights the importance of monitoring and treating elevated blood glucose in acutely ill patients receiving steroid therapy.

## Introduction

Diabetic ketoacidosis (DKA) is a life-threatening complication of diabetes mellitus (DM). Although more classically seen in patients with type I DM, patients with type II DM (T2DM) may also develop the condition in the appropriate setting [1]. DKA typically presents as elevated blood glucose 250-600 mg/dL, bicarbonate < 18 mmol/L, an elevated anion gap > 10 mEq/L, moderate to large urine ketones, and elevated serum beta-hydroxybutyrate with acidosis [1]. The pathophysiology of DKA is well known. In individuals without DM, pancreatic beta cells take up serum glucose and produce insulin; however, in DKA, a relative insulin deficiency leads to increased gluconeogenesis, production of glucose, and lipolysis leading to ketosis and subsequent metabolic acidosis [1]. Known to be linked to 16% of deaths related to diabetes, DKA can be a life-threatening condition [2]. Significant morbidity, including cerebral edema, and in some cases death, is not uncommon [2]. In the inpatient setting, clinicians must be aware of potential triggers and early signs of DKA, and more importantly, understand and promptly address iatrogenic causes. 

Steroids are a medication class commonly used in the inpatient setting indicated to treat many conditions, including but not limited to, chronic obstructive pulmonary disease, asthma exacerbations, rheumatological diseases, and inflammation secondary to acute infections [1]. While steroids are often used in this acute setting, protocols for measuring blood glucose should be put in place to minimize morbidity and prevent prolonged length of stays for those at risk of clinically significant elevated blood glucose levels, especially in those patients without an ordered diet and frequent blood glucose checks [3]. Several studies have outlined the incidence of steroid-induced DKA, creating another layer of complexity to patient management to avoid hospital stays [3]. Here, we present a case of a 50-year-old gentleman with a known history of T2DM who developed DKA less than a day after receiving IV dexamethasone to reduce swelling during a bedside incision and drainage of a peritonsillar abscess in the absence of insulin coverage [3].

## Case presentation

**Figure 1:**
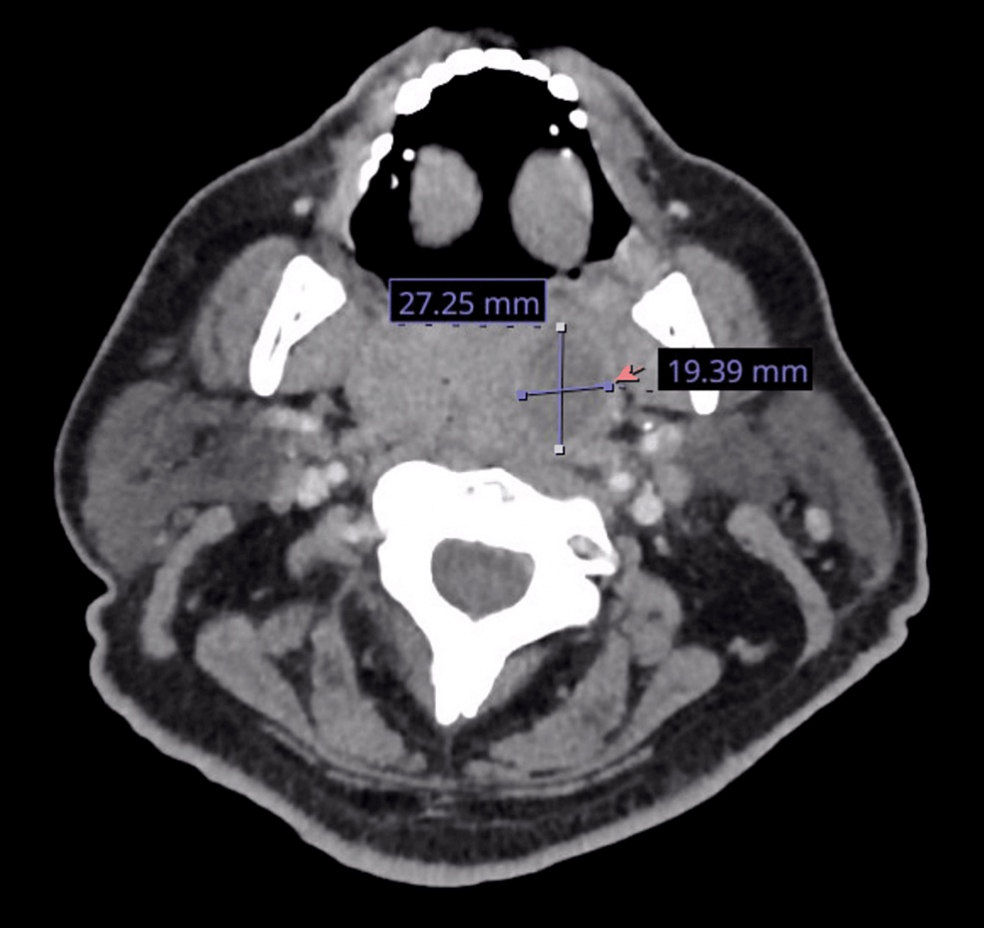
Soft tissue neck computed tomography demonstrating a large peritonsillar abscess measuring 27.25 mm x 19.39 mm (red arrow)

The patient was a 50-year-old male with a known history of uncontrolled T2DM with a hemoglobin A1C of 11, hypertension non-adherent to home-medical therapy, and class three obesity (BMI 44), who initially presented to the hospital with a three-day history of sore throat, mild difficulty swallowing, associated shortness of breath, and progressively worsening neck swelling. Per the patient, he had not visited his primary care physician regularly and was non-compliant with outpatient medications metformin, insulin lantus, and lisinopril. Due to the patient’s endorsements of difficulty in swallowing and breathing with associated neck swelling, computed tomography soft tissue neck was performed and revealed evidence of palatine tonsillitis with a large peritonsillar abscess (Figure 1).

Despite mild dysphagia and shortness of breath, the patient was able to protect his airway and did not require intubation. Otolaryngology performed initial drainage of the peritonsillar abscess at bedside and administered 10 mg of IV dexamethasone. IV Unasyn was started for antibiotic coverage and the patient was transitioned to 8 mg dexamethasone every eight hours to reduce subsequent swelling. The abscess culture grew *Streptococcus pyogenes* and *Streptococcus sanguinis*. 

The patient tolerated the incision and drainage without complication; however, he was instructed not to eat due to dysphagia. At this time, the patient had received a total of 18 mg IV dexamethasone without insulin coverage. Frequent glucose checks were not performed as the patient was without a diet order. The following morning, the patient was re-evaluated at the bedside. He was without acute concerns; however, there was a high suspicion for the development of DKA in the setting of elevated blood glucose, significantly elevated anion gap, and the presence of urinary ketones in the setting of T2DM (Table 1). The patient was promptly initiated on a DKA insulin protocol including electrolyte replacement, an insulin drip, and D5W (dextrose 5% in water) fluid. After anion gap closure, the patient was transitioned to subcutaneous insulin. During his hospital course, dexamethasone was continued for 48 hours along with Unasyn coverage. The patient was able to tolerate food, transitioned to oral Augmentin, and discharged safety home. 

**Table 1:** Relevant laboratory values at admission and throughout hospitalization Development of diabetic ketoacidosis suspected due to elevated anion gap and urinary ketones in the setting of type 2 diabetes mellitus. pCO2: partial pressure of carbon dioxide

Measured Variable	Initial Laboratory Values	Subsequent Laboratory Values	Reference Range
White Blood Count	19,000/uL	9,000/uL	4,000-11,500/uL
Bicarbonate	22 mEq	12 mEq	22-29 mEq
Glucose	353 mg/dL	489 mg/dL	80-180 mg/dL
Anion Gap	12 mEq/L	23 mEq/L	4-12 mEq/L
Beta-Hydroxybutyrate	-	8.0 mmol	<0.6 mmol
pH	-	7.19	7.35-7.45
pCO2	-	33 mmHg	30-40 mmHg

## Discussion

DKA is a life-threatening condition with a reported mortality rate of 0.2-2.5%; however, early recognition and treatment can improve outcomes [1]. Associated complications include hypokalemia, cerebral edema, rhabdomyolysis, and respiratory failure requiring intubation [1]. Although DKA typically presents in patients with type 1 diabetes, cases have also been presented in those with T2DM and it is even more common in those with severe beta-cell dysfunction, often labeled as "ketosis-prone" [1,4]. Outlined by the life-threatening complications above, our case highlights the importance of recognizing iatrogenic causes for DKA and raises the question of introducing systemic protocols in place for recognizing those at high risk of steroid-induced DKA. Difficulties often arise after steroid initiation due to the unpredictable nature of blood glucose, especially due to the difference in potency between steroid classes [5]. Rayman et al. recommended the co-administration of Neutral Protamine Hagedorn (NPH) insulin in the setting of dexamethasone initiation, such as in the current, at a dosage of 0.3 IU/kg/day, with two-thirds being initiated in the morning and the remaining in the evening [5]. Ultra-long-acting insulin regimens have also been recommended, but more studies are needed to explore this therapy [6].

A review of the literature revealed a correlation between steroid use and DKA development, even in patients with well-controlled T2DM and patients without diabetes [3]. Alakkas et al. described an increased frequency of hospitalized patients developing hyperglycemia in the setting of high-dose steroids, and in about 2% of cases, leading to a new-onset diagnosis of diabetes [7]. An increased vigilance should be suspected in specific patient populations requiring long-term steroid therapy [7]. Prior studies have also attempted to identify risk factors that may predispose patients to developing ketoacidosis in the setting of glucocorticoid therapy. Mondal et al. found a significant association between the development of ketoacidosis in individuals with a BMI < 25.56 kg/m^2^, hemoglobin A1c > 8.35 mg/dL, and IL-6 levels >50.95 pg/mL [8]. Because these patients are at higher risk, prompt treatment of their hyperglycemia and likely initiation of basal insulin may help prevent the development of DKA [7].

The association of DKA and abscesses in patients with uncontrolled diabetes has been described in the literature; however, multiple presentations of DKA exist [9]. Shizumo et al. described a similar uncontrolled diabetic patient with an acute complaint of a methicillin-sensitive *Staphylococcus aureus* (MSSA) back abscess who developed DKA after an untreated hyperglycemic state [9]. Differing from prior case reports, the current case highlights a patient’s dietary status for consideration in the monitoring of blood glucose and non-compliance with home medications, especially in those patients requiring steroid therapy. In the present case, the concern of airway compromises due to the patient's abscess led to our patient maintaining an NPO (nil per os/nothing by mouth) status in the setting of an emergent procedure. Because of this, basal-bolus insulin was not ordered and the patient was started on sliding-scale insulin with plans to adjust the regimen based on blood glucose readings. This is typical in patients not receiving food; however, especially in this patient's requiring steroid therapy, a basal insulin regimen should be considered. Our case also highlights the importance of close monitoring of blood glucose levels, especially for those with uncontrolled diabetes non-compliant with home medication regimens. Because of their medication non-compliance, these patients have a propensity to develop severely uncontrolled blood glucose levels while hospitalized. For future cases, consideration of starting a non-compliant patient on their home insulin regimen may be suitable. An additional case specifically compared outcomes of deep neck abscesses, such as that in the current case, in those with and without diabetes [10]. Results showed that those with diabetes were kept in the hospital for a longer period of time due to complications. Future studies should be considered to explore prolonged hospitalization and the development of DKA in this same patient population. 

## Conclusions

The current case highlights the importance of closely monitoring high-risk patients with diabetes mellitus receiving steroid therapy, especially in those presenting with acute infections. This case highlights the management of a peritonsillar abscess inciting DKA in a high-risk patient population, which is a unique inciting event of DKA not currently described in the literature. This case also highlights the importance of heightened vigilance for the development of DKA in patients with an NPO dietary status, as current protocols in place do not trigger frequent blood glucose checks. We propose emergency departments and admitting teams develop protocols to closely monitor blood glucose and pH levels despite a patient’s dietary status, specifically in those patients exposed to steroids. We highlight the identification of DKA in such a patient and emphasize the importance of initiating and escalating insulin therapy in this patient with clear indicators of DKA. 
